# Developing a tool to measure health worker motivation in district hospitals in Kenya

**DOI:** 10.1186/1478-4491-7-40

**Published:** 2009-05-20

**Authors:** Patrick M Mbindyo, Duane Blaauw, Lucy Gilson, Mike English

**Affiliations:** 1 Kenya Medical Research Institute Centre for Geographic Medical Research Coast-Wellcome Trust Collaborative Programme, Nairobi, Kenya; 2 Centre for Health Policy, School of Public Health, University of the Witwatersrand, Johannesburg, South Africa; 3 Health Policy Unit, London School of Hygiene and Tropical Medicine, London, UK; 4 Department of Paediatrics, University of Oxford, John Radcliffe Hospital, Oxford, UK

## Abstract

**Background:**

We wanted to try to account for worker motivation as a key factor that might affect the success of an intervention to improve implementation of health worker practices in eight district hospitals in Kenya. In the absence of available tools, we therefore aimed to develop a tool that could enable a rapid measurement of motivation at baseline and at subsequent points during the 18-month intervention study.

**Methods:**

After a literature review, a self-administered questionnaire was developed to assess the outcomes and determinants of motivation of Kenyan government hospital staff. The initial questionnaire included 23 questions (from seven underlying constructs) related to motivational outcomes that were then used to construct a simpler tool to measure motivation. Parallel qualitative work was undertaken to assess the relevance of the questions chosen and the face validity of the tool.

**Results:**

Six hundred eighty-four health workers completed the questionnaires at baseline. Reliability analysis and factor analysis were used to produce the simplified motivational index, which consisted of 10 equally-weighted items from three underlying factors. Scores on the 10-item index were closely correlated with scores for the 23-item index, indicating that in future rapid assessments might be based on the 10 questions alone. The 10-item motivation index was also able to identify statistically significant differences in mean health worker motivation scores between the study hospitals (p < 0.001). The parallel qualitative work in general supported these conclusions and contributed to our understanding of the three identified components of motivation.

**Conclusion:**

The 10-item score developed may be useful to monitor changes in motivation over time within our study or be used for more extensive rapid assessments of health worker motivation in Kenya.

## Background

There has been an upsurge of interest in human resources required to deliver health care in low-income settings as part of the drive to achieve the Millennium Development Goals. Much of the attention, including in Kenya, has focused on the inadequate numbers of health care workers and their inequitable distribution [1-3]. However, it is increasingly appreciated that attention must also be paid to health worker performance [4-8]. Many factors – ranging from available physical infrastructure to an individual's highly personal values – influence the performance of health professionals [6]. Many of these factors influence performance through the health worker's motivation, where motivation is defined as an individual's degree of willingness to exert and maintain an effort towards attaining organizational goals [9].

Although it is likely that motivation influences performance directly and mediates or modifies the effect of interventions aimed at changing performance [6], there are few studies on its influence on practice change in health workers in low-income settings [6,10]. The existing studies have focused predominantly on determinants of motivation, with less literature focusing on conceptualizing and measuring worker motivation [11-14]. One way of measuring motivation is delineated by Franco et al.'s model [9] (based on Kanfer 1999) [11], which divides determinants of motivation into "will do" (i.e. adoption of organizational goals) and "can do" components (i.e. mobilization of personal resources to attain joint goals) that operate at individual, organizational and societal levels [9,11]. Motivational outcomes are viewed to be the net results of the interaction between the "can do" and "will do" components of motivation [9,11] and are the main focus of this study. This approach views worker motivation to be the result of the interaction of individuals and organizations, rather than an attribute of either alone [11].

To measure motivation, one can either use subjective (asking workers their perceptions of motivation and what influences it) or objective measures (directly observing issues such as timeliness, or checking attendance records) [11,14]. While objective measures of assessment are thought to be better than subjective ones, indicators such as absenteeism would be very difficult to apply in our circumstances where, for example, records are poor or non-existent. On the other hand, directly asking workers whether or not they are motivated risks introducing response bias (i.e. respondents answering questions in the way they think the questioner wants them to be answered, rather than according to their true beliefs).

With these considerations in mind, we assessed motivation by examining underlying issues grouped around relevant outcome constructs such as job satisfaction, general motivation, burnout, work quality, absenteeism or turnover [11,14] that collectively reveal levels of motivation. Studies tackling this issue in developing country settings have predominantly used qualitative methods, as shown by work done in Ethiopia [15], Tanzania [12] and North Viet Nam [16], with a mixed-methods approach being used in Mali [13].

We therefore explored the possibility of using a self-administered questionnaire to measure motivation among Kenyan health workers in eight hospitals. These hospitals are taking part in a study evaluating the implementation of guidelines intended to improve paediatric care [17,18]. A suitable measure of motivation would allow us to examine motivation as a contextual influence on the ability of the intervention to improve health worker practice in district hospitals in Kenya.

Furthermore, a tool that could be rapidly administered to large numbers of staff and in large numbers of facilities might allow motivational scores to be used to explore, at least in part, the association of motivation with health system performance. While such large-scale studies would provide the ultimate test of the validity of a scoring tool, the present work focuses on the process of tool development.

## Methods

### Overview of the study

This work was part of a set of baseline surveys undertaken for a larger intervention study being conducted in selected Kenyan district hospitals. The main study seeks to investigate the degree to which the quality of paediatric inpatient care in these sites can be improved and is described in detail elsewhere [17,18]. The intervention has been developed with the Ministry of Health (MoH) and is being delivered over 1.5 years to four intervention hospitals and in a much more limited fashion to four control hospitals (i.e. five-day intensive training with supervision as well as written and verbal feedback, versus 1.5 days of lectures and written feedback but without supervision) [17,18]. The clinician's uptake of and adherence to practice guidelines is being assessed within each hospital; those findings will be reported in due course.

Our aim here was to develop a tool (i.e. a simple motivational index) to measure motivation that was as parsimonious as possible [11] and that could be used to describe motivation in more rapid, repeated assessments in the future. We reasoned that a quantitative or semiquantitative tool for measuring motivation, particularly one that can be easily administered, would allow us to explore both the influence of baseline motivation in a hospital on the response to the intervention and the interaction between motivation and delivery of the intervention over time. Qualitative work was undertaken as part of the larger study [17,18] to explore in-depth issues around motivation that would affect the implementation of the practice change intervention. In this paper, qualitative work was undertaken to assess the relevance of an initial longer set of questions and to explore the face validity of the tool [19].

### Tool development

The starting point for potential constructs and questions to be included in the self-administered questionnaire (SAQ) was a questionnaire prepared for South African nurses [20], in turn based on earlier work in Georgia and Jordan [11,21]. Additionally, literature was reviewed on studies of motivation of health workers, taking particular note of those we considered most relevant to the Kenyan situation [9,11,13,14,20,22-24]. From these sources, and from a review of studies that used motivation theory in health [10], we identified constructs we felt could be categorized as likely outcomes of motivation.

We therefore began by including a broad range of constructs considered potentially important, while aiming to include at least three questions per construct. This resulted initially in 17 potential constructs divided into two broad categories representing determinants (10 constructs) and outcomes (seven constructs) of motivation. As a result, the initial, pilot SAQ had 71 questions answered on a five-point Likert scale ranging from "strongly " to "strongly disagree" The questionnaire also included a "Don't know" response for each question. Questions were randomly assorted, with about 40% worded negatively to avoid response-set bias.

### Pilot-testing

The SAQ was pilot-tested in two non-study public hospitals in Kenya to test for clarity of questions and to gain preliminary insight into the SAQ's construct validity. Fifty-five pilot questionnaires were received and analysed, first by checking the direction, magnitude and variability of the responses. Second, correlation of items within a construct were tested with Cronbach's alpha, evaluating the degree to which responses within sets of questions supported their theoretical grouping.

Questions not performing as expected were reworded for clarity. In some, this meant reversing the negative wording that seemed to be responsible for the question's poor performance, because respondents misunderstood the intended meaning. This process was complemented by concurrent qualitative work that helped to identify potentially sensitive questions [25].

Based on the pilot results, a SAQ with 17 constructs and 66 questions was finally developed. The focus of the present report is restricted to data from the seven constructs and 23 questions relating to motivational outcomes that were used to develop a score of motivation (Table 1).

**Table 1:** Motivational outcome constructs and questions

Construct	Questions	Mean score (1–5)
General motivation	These days, I feel motivated to work as hard as I can	2.77
	I only do this job so that I get paid at the end of the month	4.01*
	I do this job as it provides long term security for me	3.54*

Burnout	I feel emotionally drained at the end of every day	2.79*
	Sometimes when I get up in the morning, I dread having to face another day at work	3.39*

Job satisfaction	Overall, I am very satisfied with my job	3.42
	I am not satisfied with my colleagues in my ward	3.83*
	I am satisfied with my supervisor	3.62

Intrinsic job satisfaction	I am satisfied with the opportunity to use my abilities in my job	3.79
	I am satisfied that I accomplish something worthwhile in this job	4.17
	I do not think that my work in the hospital is valuable these days	4.05*

Organizational commitment	I am proud to be working for this hospital	3.93
	I find that my values and this hospital's values are very similar	2.95
	I am glad that I work for this facility rather than other facilities in the country	3.23
	I feel very little commitment to this hospital	3.89*
	This hospital really inspires me to do my very best on the job	2.97

Conscientiousness	I cannot be relied on by my colleagues at work	4.42*
	I always complete my tasks efficiently and correctly	3.98
	I am a hard worker	4.50
	I do things that need doing without being asked or told	4.28

Timeliness and attendance	I am punctual about coming to work	4.27
	I am often absent from work	4.50*
	It is not a problem if I sometimes come late to work	4.16*

* The scale for negatively worded questions was 1(strongly agree) to 5 (strongly disagree). Thus a high score shows disagreement with a negative statement and is therefore suggestive of higher motivation.

### Sampling and data collection

On the basis of previous survey experience [26], whole-population sampling was not considered feasible in the study setting of rural Kenyan hospitals. A sample of 30% (i.e. 90 staff members from each hospital) and a total sample size of 720 was considered statistically adequate for these analyses. All baseline data were collected by identically trained survey teams on-site (two weeks per site) who distributed and collected SAQs to staff members expected on duty for more than two days during the survey period. Staff members working in paediatric areas or other areas but with regular contact with sick children in their day-to-day work were preferentially targeted for SAQ distribution, because the main intervention was aimed at improving paediatric care.

All qualitative data were collected by the lead author during one-week visits to each hospital made after the departure of the main survey teams [27,28]. These visits were conducted from late August to September 2006 prior to any intervention. A purposive sampling approach was used to select participants to be interviewed. The hospital CEO, administrator, matron and ward in-charges and clinicians (doctors and clinical officers) were chosen as key informants, as they are few in number but have a wide knowledge of hospital operations due to their job functions. As such, an effort was made to interview all present during the one-week visit. Focus group discussions (FGDs) were conducted among nurses (especially in maternity and child health sections), as they form over 50% of the clinical staff in the hospitals. Focus group discussions took place mainly in the late afternoons, when workloads were considerably reduced.

### Data analysis

Data were double-entered by means of a purpose-designed Microsoft Access 2003 interface. The principal investigator carried out verification, including checking of missing data [29]. After this, quantitative data were analysed with STATA 9.2. Likert-scale responses were entered as a score of 1 to 5. A score of 5 represented the statement "strongly agree" for positively-worded questions, while negative questions were coded in the opposite direction, so that a score of 5 represented "strongly disagree".

Responses to individual questions were examined by means of frequency distributions, mean and median scores and examining whether the direction of response was as anticipated and consistent with responses within and across constructs – especially for negatively-worded questions. The relationships between responses to questions forming a construct were examined using Pearson's and rank correlation (coefficients above 0.5 were deemed to be good) and as a set using Cronbach's alpha.

Factor analysis was used to identify groups of interrelated variables. Underlying constructs, also known as latent factors, were deduced from the correlations between the measured variables of the questionnaire and provided a basis for data reduction and the development of a new simplified index.

Qualitative data were transcribed and imported into NVIVO7, where identification of themes followed the conventional coding process [30]. The results were combined and subsequently collated into relevant, larger thematic categories to improve explanatory ability.

### Ethical issues

Ethical clearance for the broader study was obtained from Kenya's National Ethics Review Committee, and permission was sought from the heads of each hospital. Oral assent was sought for administering a SAQ; written consent was sought for interviews and FGDs from the study participants.

## Results

### Quantitative data

In total, 720 SAQs were distributed and 684 (95.0%) were returned. Of the 684 SAQ's returned, one had almost no recorded responses and another 15 (evenly distributed across the eight sites) had > 20% missing responses; all of these were subsequently dropped from further analysis. In the 668 remaining SAQs, out of the 44 088 total possible responses (668 × 66), 89 were missing (0.2%) and 621 were "Don't know" responses (1.4%). Factor analysis requires complete data, and thus these analyses could have been restricted to a set of 427 perfectly complete SAQs. As this would have resulted in a large number of valid responses' being dropped, we imputed for each "Don't know" response a neutral response (score 3) to create a complete dataset of 634 SAQs for factor analysis (34 SAQs remained excluded from factor analysis because of genuinely missing responses).

We compared the factor analyses of the original dataset with the one using imputed values. As there were no material differences between the results and because imputing missing data made maximum use of the actual responses provided, we report here only the results from the analyses incorporating imputed data.

There were differences in the profiles of respondents between hospitals, with considerable variation in the proportion who were clinicians, the proportion who worked in paediatric areas (as defined above) and the proportion who were female (Table 2). These differences between hospitals reflect both their difference in total and departmental size – smaller hospitals have fewer clinicians – and perhaps the sampling approach (the data collection process focused on staff members primarily working in paediatric areas, where the intervention was to be implemented). Overall there were more female (58.9%) respondents than male (41.1%), which concurs with the findings of the 2004 MoH Human Resource Mapping exercise, which found more female workers (52.7%) than male (47.3%) in Kenya's health workforce [5]. In terms of workplace, the main non-paediatric areas represented were adult inpatient services (17.6%) and laboratory and radiology departments (6.0%).

**Table 2:** Respondent characteristics for the eight study hospitals

	Hospitals										
				
Respondent characteristics	H1	H2	H3	H4	H5	H6	H7	H8	**Total** **(%)**	**X**^2^	**P value**
Gender (female %)	59.8	72.4	49.3	69.1	51.8	47.9	61.0	58.4	58.9	17.6	0.014

Paediatrics (%)	39.5	69.6	45.0	50.0	32.5	60.3	48.8	54.8	49.6	30.4	< 0.001

Clinicians (%)	77.5	83.8	73.0	70.9	67.9	52.6	72.4	73.1	71.6	21.6	0.003

The mean score for each of the original 23 questions is shown in Table 1. The means have been calculated with the scoring of negative questions reversed (as described above) so that higher means indicate higher motivational outcomes whatever the wording of the original question. The highest mean scores were for questions 19 and 22, indicating that the majority of respondents strongly agreed that they were hard workers and disagreed that they were often absent from work. Nevertheless, the lowest mean score was for question 1, which suggests that many participants would describe themselves as demotivated.

### Analysis of motivational outcomes

We used both inter-item correlation and factor analysis to evaluate patterns in the responses of respondents. Through correlation, we examined how questions performed within and between constructs. All 23 questions taken together as a single index of motivation had a Cronbach's alpha of 0.75. Individual constructs performed less well, with Cronbach's alphas ranging from 0.36 to 0.64 demonstrating, in part, the relationship between increased number of questions per construct and higher Cronbach's alpha scores.

Factor analysis, on the other hand, showed that three latent factors explained the majority of the variance in the data. These results (not shown) suggested that the motivational outcome questions in the SAQ related to three main underlying themes, rather than the seven originally proposed constructs. Questions related to the organizational commitment loaded onto the first latent construct (39.5% of variance). The second latent construct (28.7% of variance) gathered together questions around conscientiousness, timeliness and attendance, while questions associated with general motivation, job satisfaction and burnout loaded onto the third latent construct (23.3% of variance).

### A simplified index of motivation

Although we could have used a motivation score with all 23 items, we aimed to be as parsimonious as possible and produce a more simplified index to facilitate subsequent data collection. To reduce the number of questions needed, we initially identified items with the highest factor loadings on the three main latent factors and then adhered to the following principles: balancing the number of questions from each construct, dropping those that correlated least well, and examining inter-item correlation and Cronbach's alpha to include questions that performed well within their constructs and for the overall index. This resulted in selection of 10 primary questions whose factor analysis results are shown in Table 3. The 10 questions grouped into the same three underlying factors, but factors 2 and 3 were now reversed. As can be seen in Table 3, the weightings for each question were fairly similar. Therefore, to simplify subsequent use of the index, we proposed using an equally-weighted index using these 10 questions. Using the original scoring of 1 to 5 for each question meant that the index would have a potential range from 10 to 50, with a midpoint of 30.

**Table 3:** Factor analysis of the 10-item motivation index (rotated factor loadings and unique variances)

Variable	Factor 1: *Organizational commitment*	Factor 2: *Job satisfaction*	Factor 3: *Conscientiousness*
No motivation		0.4478	

Very satisfied with job		0.5928	

Satisfied with opportunity to use my abilities in my job		0.5348	

Job makes me feel good about myself	0.4199		

Proud to be working for this hospital	0.5420		

Glad to work for this facility than others in the country	0.5701		

Hospital inspires me to do my very best on the job	0.4837		

I always complete my tasks efficiently and effectively			0.5224

I am a hard worker			0.5322

I am punctual about coming to work			0.4812

Blanks represent |loading| < 0.4

We confirmed that the simplified 10-item index was comparable to the original index by calculating the Pearson's correlation coefficients of individual's scores (Table 4). We found a strong correlation of 0.9608 (p < 0.0001) between our shorter, equally-weighted 10-item score and the score for all 23 questions. Table 4 also confirms that the score using equally weighted questions is very similar (r = 0.9821) to the more accurate score derived from factor analysis.

**Table 4:** Correlation between different motivation scores

	23-Item Score (Factor loadings)	10-Item Score (Factor loadings)	10-Item Score (Equally weighted)
23-Item Score (Factor loadings)	1.0000		

10-Item Score (Factor loadings)	0.9798 (p < 0.001)	1.0000	

10-Item Score (Equally weighted)	0.9608 (p < 0.001)	0.9821 (p < 0.001)	1.0000

### Using the score

Using the simple, equally weighted, 10-item index, we calculated the mean motivational score for each study hospital (Table 4). The mean motivational scores for the eight hospitals ranged from 35.9 (H6) to 39.3 (H2), indicating generally positive motivation levels, since the means were all above 30. Overall, the differences in mean motivation scores between hospitals were statistically significant (ANOVA, p < 0.001), but multiple comparisons testing revealed that the differences were primarily between the three lowest-scoring hospitals (H6, H1 and H7) in comparison to H2, the highest scoring hospital (Bonferroni test, p < 0.05).

Clearly the score based on 10 items represents a summarization of item scores from the three latent factor groupings. In Table 5 we also present the mean score per item for each of the three latent factor groupings. In general, the high-scoring hospitals overall appear to have higher scores in comparison with other hospitals for each latent factor, although there is some variability in rankings when comparing the overall score with the factor-specific scores. Interestingly, the factor-specific mean scores show consistency within a factor grouping, with hospitals having relatively similar absolute mean scores and relatively small standard deviations in all cases. However, there is variability in the absolute values of the means between factor groupings. Thus latent factor 2 (job satisfaction) has consistently low scores in each hospital, followed by factor 1 (organizational commitment), while factor 3 (conscientiousness) is higher than both. The higher scores for factor three items might be because questions in factor 3 reflect on them as individuals (a form of positive response bias), while the lower job satisfaction and organizational commitment scores perhaps reflect more on the "system".

**Table 5:** Mean summary 10-item motivation score (minimum 10, maximum 50), from highest to lowest, and mean item scores for each latent factor by hospital

Hospital	Mean 10-Item Motivation Score	Standard Deviation	Mean Latent Factor 1	Mean Latent Factor 2	Mean Latent Factor 3
H2	39.31	4.83	3.80	3.67	4.37

H3	37.93	5.33	3.74	3.47	4.20

H4	37.09	5.29	3.49	3.34	4.35

H8	36.62	4.87	3.57	3.39	4.05

H5	36.46	5.43	3.49	3.18	4.27

H7	36.29	5.63	3.33	3.39	4.27

H1	36.04	6.27	3.55	3.02	4.25

H6	35.91	5.88	3.40	3.20	4.23

Average	36.94	5.54	3.55	3.33	4.25

We used multiple regression to explore whether or not the differences in characteristics of the hospital respondents at baseline (see Table 2) explained the difference in motivation scores between hospitals. The results of this regression are shown in Table 6. Certain differences between hospitals persisted in multivariate analysis – H2 was significantly higher, and H1 had significantly lower motivation scores than average. Female health workers, working in paediatrics and being a clinical officer (CO) did not significantly affect motivation, but medical officers (MOs) had statistically lower scores, and non-clinical workers were more motivated than average. Although the overall model was statistically significant, it had very limited explanatory power (R^2 ^= 0.056). This is in contrast to a regression model that included the motivational determinants from the original questionnaire (results not shown) that had an R^2 ^of 0.551.

**Table 6:** Multiple regression model of motivation score

Variable		Coefficient	SE	p Value
Constant		36.847	0.450	p < 0.000

Hospitals	H1	-1.475	0.623	0.018

	H2	2.580	0.604	0.000

	H3	0.661	0.580	0.255

	H4	0.101	0.620	0.870

	H5	-0.455	0.578	0.432

	H6	-1.086	0.579	0.061

	H7	-0.487	0.584	0.405

Gender	Male	-	-	-

	Female	-0.250	0.549	0.649

Paediatric staff	No	-	-	-

	Yes	-0.560	0.483	0.247

Staff category	CO	0.240	0.508	0.637

	MO	-1.948	0.784	0.013

	Non-clinical	1.090	0.455	0.017

Overall model: p = 0.0008, R^2 ^= 0.056

Dummy coding used for Gender and Paediatric variables so that coefficient is in comparison to reference group. Effect coding used for Hospital and Staff category variable so that coefficient compares group to overall mean.

### Qualitative results

Our use of qualitative approaches was two-fold: to improve SAQ development and to help us understand the SAQ results. With regard to SAQ development, qualitative work improved the tool, as explained previously in this paper, showing unclear questions that needed rephrasing. In terms of explanatory ability, our understanding of the data captured by the SAQ was supported or enhanced by qualitative data. These findings will be discussed further in the next section.

## Discussion

### The motivation score

Based on existing conceptual and empirical work, we developed an SAQ to assess motivation in hospital-based Kenyan health workers. Additionally, a comparison of the quantitative and qualitative results was made to help understand the motivation score. Very high response rates were achieved by research staff as a result of combining SAQ administration with other hospital survey tools [17,18]. By means of factor analysis we identified 10 questions, representing three latent factors, that appeared suitable for use as a rapid tool for quantitative assessments of motivation.

Qualitative data and reflection on observations made by the PI in this study during fieldwork suggest that the simplified index appropriately indicates variable levels of motivation between hospitals, showing that the score could be an important component of motivation analysis. For example, the qualitative analysis suggested that management in the hospital with the highest mean motivational score (H2) was considerably better than the average, and supported the conclusion that motivation was highest in that hospital. However, it should be remembered that neither hospital staff nor hospitals were randomly selected, and thus hospital mean scores should be interpreted cautiously. This does not, in our opinion, detract from the usefulness of the index or the finding that the index was able to differentiate study hospitals according to their workers' reported motivational levels.

Further, while reflecting variability between sites, care should perhaps be taken not to over-interpret the absolute values of the index score. While the score appears to suggest that on average people were slightly more positive than negative, with a mean of 36.9 out of a possible 50 points, these data still suggest the need for improvements in motivation of health workers, a view strongly reinforced by the qualitative work (in preparation).

When scores within individual item groupings resulting from factor analysis are examined, some consistency in hospital ranking is observed, although there is a suggestion that scores are quantitatively lower (satisfaction) for some factor groupings than others (conscientiousness), although this may be explained by response biases. Additionally, the score is unable to show nuances such as the role of leadership in improving worker motivation or the importance of clear communication between hospital management and staff, which were clearly highlighted in the qualitative work. This being so, there is need for complementary qualitative approaches that improve the researcher's ability to explain such findings [19].

### Qualitative and quantitative results

With regard to motivational outcomes, the first latent factor grouped together questions around organizational commitment with relatively high mean scores (Tables 1 and 5). Qualitative data reveal that all health workers were attracted to health care work by some aspect of public service or the altruistic nature of health care provision, suggesting a strong sense of attachment subsequently reinforced by professional training. However, an inability to do their work due to constraints such as high workloads, old buildings and lack of drugs and non-medical supplies caused dissatisfaction with health care work. This led to outcomes such as: "...staff experience [ing] burnout resulting in poor attitudes to patients and work." [*Matron, H1*]. Other outcomes include shirking duties, moonlighting, laxity at work or efforts to change jobs, which echo findings in Ethiopia [15] and Malawi [31]. As such, the SAQ responses in the first latent factor appear related more to workers' commitment to the ideals of health work as a profession and less perhaps to actual, individual behaviours such as acceptance of organizational goals, working practices or intention to remain in the organization [32].

However, organizational commitment could be positively modified by efforts of senior management, as shown by qualitative data. In one site (H3) where a senior administrator was actively involved in running the hospital, motivation was reportedly high despite the site's having small buildings and shortages of medical supplies and equipment. Where this did not happen, a worker felt that: "a little effort by the med sup [medical superintendent] to have, say, an annual process of recognising staff say, Nurse, CO, Doctor etc would really help staff to realise that the management was watching what they do and would reward good work" [*Senior Clinical Officer, H4*]. Thus, the perception that some senior management officers did not appreciate or recognize their staff [4,33,34] might have the effect of lowering a person's level of conscientiousness, resulting in behaviours such as not turning up to work.

As to the second factor, SAQ data suggest relatively high levels of self-reported conscientiousness, timeliness and attendance, which should be reflected by high levels of productivity in the hospitals (see Table 1). However, observations and interviews in the same sites show a different picture: "They [hospital staff] have started clocking in as a result of the laxity, though, even if they come on time, it is not known if they are working well or not" [*Acting Hospital Matron, H4*]. This dichotomy is not surprising, since the SAQ shows the health workers' positive subjective assessments of their motivation, with which their supervisors do not agree. This clearly shows the value of using both subjective and objective measures of motivation for purposes of triangulation, as well as validating the results acquired.

Qualitative data also suggested that workers did not differentiate between general motivation and burnout issues as separate influences over their motivation, which is consistent with the grouping of the two constructs into the third latent factor by factor analysis. Interestingly, SAQ responses with reference to the third latent factor generally revealed low mean scores across all eight sites (Table 1), perhaps because high workloads, shortages of qualified staff and lack of equipment and medical supplies are commonly encountered.

### Limitations of our study

Methodologically, the exploration of a multidimensional theoretical construct (such as motivation) ideally requires a substantial number of questions yielding a high Cronbach's alpha score for a question set. Since we did not have prior experience of testing motivation in the Kenyan context and because we wished to produce a questionnaire of manageable length, we chose to have a relatively broad range of constructs with relatively few questions per construct. This is because of the general feeling that very long questionnaires are less well completed which means that there are trade-offs in tool design in terms of length, number of constructs and number of questions per construct.

Additionally, respondents' perceptions of what constitutes an acceptable answer, how their answers reflect on them, or what they think the investigator wishes to hear might have influenced the way they answered our questions [25]. To overcome response-set bias, we worded half the questions positively and the other half negatively. We found, during the pilot phase, that positive questions seemed to perform better in our context, which may explain why all questions included in our 10-item score are positive. This is similar to other findings from lower-income settings reported by Franco and her colleagues [21]. Here, the context of implementation of the project also needs to be considered. The baseline survey was a year after resumption of duties by health workers who were affected by a general health workers' strike that took place in June 2005, which we believe may have influenced their responses negatively.

Our analysis was also potentially affected by a number of truly missing responses and inclusion of a "Don't know" option. This option was included as a result of pilot work suggesting that some participants did not want to answer more sensitive questions or sometimes did not know how to answer the question. This possibly reflects the difficulty in adapting questions developed in other settings [11]. However, results indicate that the "Don't know" response was made in only 1.4% of possible occasions. Thus, although the literature shows that the inclusion of the "Don't know" option may substantially lower the number of respondents offering their opinions [25], in our case it would seem to have made little difference to our overall interpretation. Conversely, the limited use of this option would also indicate that a "Don't know" response could be omitted in future questionnaires.

This study could have been improved by collecting SAQ data from a simple random sample of each hospital's health care staff or including only staff working in paediatric areas, at whom the intervention is primarily intended. However, while a different study sample might affect the magnitude of the hospital scores, it seems unlikely that it would result in selection of a very different 10-item index.

We are also aware that there are true differences in staffing patterns between hospitals that would be reflected in a hospital summary score. One example is the proportion of interns (i.e. pre-registration clinicians completing one year of experience prior to registration), which does differ between hospitals, but that we were unable to analyse separately because there were too many missing data for that question. For these reasons, interpreting the summary scores as typical of Kenyan hospital settings should be approached cautiously. However, the working areas we focused on represent a substantial proportion of the hospital (antenatal and maternal and child health clinic; general outpatient/emergency area, maternity ward and paediatric wards) and we achieved a very high response rate (95%). Additionally, the quantitative findings were in general supported by the parallel qualitative work and the opinions of the returning survey staff that were all consistent in identifying the least and most motivated hospitals.

More generally, our results reflect responses from hospital-based health workers predominantly providing services in areas where paediatric and paediatric-related care is required, from eight Kenyan district hospitals. From this starting point, we have been able to develop a potentially rapid motivation measurement tool aimed at exploring motivation as a contextual influence on uptake of new practices. Qualitative work suggests that altruistic motives are important to Kenyan health workers, though numerous difficulties facing the provision of health care in the public sector threaten their organizational commitment and general motivation. Further, our qualitative findings are similar to those reported in the literature from other similar settings [12,15,16].

## Conclusion

There are no "gold standard" tools to measure motivation, so we set out to develop an easily administered, quantitative tool that might allow us to explore the influence of baseline motivation on the response to a hospital-based intervention to change health worker practices and, later, the interaction between motivation and delivery of the intervention over time. The value of such a score might be further examined in large-scale studies exploring the association between motivation, measured in this way, and other attributes of the health system, such as hospital performance.

The focus of the current work was, however, to develop a rapid tool (eventually of 10 items) that, through factor analysis, appears to capture motivation quantitatively. Qualitative data suggest that the questions comprising the 10-item tool approximated issues relevant to staff motivation in district hospitals. It also emphasizes the need for an understanding of the context of implementation and concurrent qualitative work to triangulate results.

The value of this 10 item tool will become apparent only with repeated field tests, which are ongoing and which will be reported in due course. While we have restricted ourselves to interpreting the tool's results at hospital level, they can be interpreted at the individual level; further work will examine the link between hypothesized determinants of motivation and the outcomes reported here.

## Competing interests

The authors declare that they have no competing interests.

## Authors' contributions

All authors contributed to and approved the final manuscript.
